# Variation Overtime among Patients of the Six Methadone Maintenance Treatment Clinics in Thai Nguyen from 2011 to 2015

**DOI:** 10.1155/2018/9081968

**Published:** 2018-08-07

**Authors:** An Thi Minh Dao, Huong Thi Thu Nguyen, Long Hoang Nguyen

**Affiliations:** ^1^Department of Epidemiology, Hanoi Medical University, Vietnam; ^2^Department of Biostatistics and Health Informatics, Hanoi Medical University, Vietnam; ^3^Vietnam Administration of HIV/AIDS Control, Vietnam

## Abstract

**Background:**

Methadone Maintenance Treatment (MMT) program's success depends on the likelihood of reducing drop-out rate and keeping patients remaining in the program. There have been neither comprehensive studies about variation among patients who have been experiencing MMT for long period nor prediction of MMT period in which the risk of drop-out would be the highest in Thai Nguyen, a northern mountainous province where the MMT was established in 2011.

**Objectives:**

To analyze variation of the MMT population through indicators of drop-out and death, re-enrolment, and retention rate in the six Thai Nguyen MMT clinics.

**Methods:**

A retrospective study by reviewing daily treatment notebooks of the six MMT clinics in Thai Nguyen to identify events of drop-out, death, reenrolment among 2,567 patients registered from 12 May 2011 to 6 September 2015.

**Results:**

Cumulative hazard of drop-out over period from the first to the fourth year of MMT treatment has an increasing trend at 0.15; 0.31; 0.46; and 0.61, respectively. The cumulative probability of re-enrolment among 740 patients who have already quit the MMT program and then returned slightly increased from 0.07 to 0.16 between the first years and the fourth year in which the highest returning rate occurred within the first 2 years after drop-out. The cumulative retention rate decreased annually and stayed at 71.7% after 4 years of running the MMT.

**Conclusions:**

MMT patients and their families should be informed and consulted about the highest risk period of drop-out and also about period when drop-out patients are most likely to reenter the MMT. Counseling adherence for patients should be conducted not only at the beginning but also during the ongoing MMT and play an extremely important role in reducing drop-out of the program while special counseling should also be reenforced for the re-enrolment patients of MMT.

## 1. Introduction

The rate of HIV infection among people who inject drugs in Vietnam has always been high in comparison with other high-risk groups such as men who have sex with men and female sex workers [1]. Opioid partial agonist such as buprenorphine is associated with misuse by intravenous route [2], leading to infective endocarditis [3] and cutaneous complications [4]. As a result, MMT is considered an effective intervention to prevent drug users from acquiring HIV because methadone use can decrease rates of heroin injection. For a long time, methadone has been used as a substitute for heroin in order to reduce the risk of HIV transmission since it is taken orally without requiring injection. Because methadone has a longer half-life than heroin, its effect is longer than heroin so that it helps distance of each user time be more longer and helps drug users have more time to stabilize their works and lives [5, 6]. However, methadone substitution is a long-term, possibly life-long process.

Literature reviews showed that injecting drug users (IDUs) who participate in the MMT have experienced variations throughout their MMT algorithm such as entering the program, receiving MMT, missing any doses or drop-out, or death at any certain time and then may return to the program later on. These variations contributed much influence to the retention rate in the MMT after a certain time period. There were large numbers of studies showing that proportion of patients remaining in the MMT is key criteria for evaluation of the success and effectiveness of that MMT program [5, 7, 8]. Besides, there were many studies indicated that drop-out rate varies from one country to others and even within a country that rate varies from one area to other ones. A study in five clinics (3 in Shanghai, 2 in Kunming) in China in 2014 revealed that 73.0% out of 319 patients have (dropped out seven consecutive days) within one year [9]. Another study in Guangdong, China, in 2015 showed that 79% of the 1,512 study participants have dropped out 14 consecutive days or more during the 7-year study period [10]. A study in Malaysia showed a 31.0% drop-out rate from 2007 to 2009 [11].

In addition to the drop-out events in the MMT patients reported, death events during the MMT is also an issue of concern. A cohort study of 3,162 Scottish drug users between January 1993 and February 2004 identified 64 drug-related deaths [12]. The greatest risk of drug-related death was in the first 2 weeks of treatment (adjusted hazard ratio 2.60, 95% confidence interval 1.03-6.56). The risk of drug-related death was lower after the first 30 days following treatment cessation, relative to the first 30 days of treatment. The risk of drug-related mortality in MMT is elevated during periods of treatment transition, specifically treatment initiation and the first 30 days following treatment drop-out or discharge [12]. A study in Malaysia revealed that of 172 MMT clinic attendees who enrolled into the program, 107 remained active after 2 years (62.2%). Of the total number of 65 who were originally reported as defaulters, 3 had passed away, 8 were transferred out to have their MMT follow-up at other health centers, 5 were incarcerated, 32 were untraceable, and 17 decided to stop [11].

Another issue which has emerged from the previous studies is the returning of the drop-out patients as the event of “re-enrolment”. Persistent cycling in-and-out of clients in MMT programs is common [10]. A study in Guangdong in 2015 indicated that of 1,194 who had ever dropped out, 81.2% reenrolled in the whole study period of which the returning rates in the first, second, and seventh year of the MMT were at 36.1%, 53.8% and 81.2%, respectively [10].

All the entering numbers, drop-out rate, and returning rate have great contribution to the rate of retention which is the proportion of patients remaining in program at the period of time. In studies with a robust number of participants, retention rates were reported differently all over the world [13, 14]. One-year retention rates reported are 74.4% in Israel [15]; 70.7% in Cambodia [16], 68.6% in Malaysia [11], 61% in Ireland [17], 57.4% in Guizhou [18], and 52% in Colombia [19]. Study in Rafsanjan, Iran, in 2016 showed the cumulative retention in treatment decreased over time in which over 50% of patients dropped out before 6 months and about one-third of them had a one-year retention [20]. In a multicenter study in Shiraz, Ardebil, Ilam, and Semnan, Iran, three- and six-month retention rates were 50% and 22.7%, respectively [21]. A study in Taiwan among 128 participants indicated that four patients withdrew from MMT within 30 days of treatment. Retention rates were 80.5% (n = 103), 68.8% (n = 88), 53.9% (n = 69), and 41.4% (n = 53) for 3 months, 6 months, 12 months, and 18 months, respectively [22].

In Vietnam, the MMT program was first tested in 2008 in Ho Chi Minh City and Hai Phong. Up until January 2016, 57 out of the 61 provinces throughout the country have been supplying MMT and providing service for 44,078 patients [23]. Reports of the two cities which firstly supplied the MMT in Vietnam showed that requirement of daily drug-intake in person at MMT clinics for long life is a major challenge for keeping patients remaining in the MMT [24].

Study in Ho Chi Minh City in 2008 showed that drop-out rate in the first six months of MMT was 1.9%; 8.8% in 2009; 10.9% in 2010; and 5.1% in 2011 while the overall drop-out rate from 2009 to 2011 was 26.6% [25]. Study conducted by FHI in Ho Chi Minh City and Hai Phong City indicated that 10.2% of patients have quit after one year and 17.7% have quit after two years [26]. An evaluation of Haiphong's MMT program in 2008 revealed that drop-out rates were low, only about 6% during the first 6 months of treatment [27]. A study in Hai Phong among 1055 clients initially enrolled from 2012 to 2014 highlighted that 10.5% have dropped out during the first year of the MMT. Among the 944 patients who were on treatment at the start of the second year, 124 (13.1%) dropped out; 819 clients were in treatment at 24 months, of whom 115 (14.0%) dropped out during the subsequent year. Over the 3-year period, a total of 350 (33.2%) clients had permanently dropped out [28].

Thai Nguyen, a northern mountainous province, is the third province in Vietnam starting to implement the MMT on 12 May 2011. By 6 September 2015, there were six MMT clinics implemented in five districts in Thai Nguyen. These were Trung Thanh, Tuc Duyen, Dong Hy, Dai Tu, Phu Luong, and Pho Yen districts supplying MMT for 2,567 patients. Up until now, there have been neither comprehensive studies about variation among patients who have been experiencing MMT for long period nor prediction of MMT period in which the risk of drop-out would be the highest. Therefore, the objectives of this study are to analyze the variation of the MMT population including drop-out and death, reenrolment, and retention rate in these six MMT clinics of Thai Nguyen province and to predict the period of MMT where the risk of drop-out is highest. This will help policy makers to develop better control measures for the MMT program.

## 2. Materials and Methods

### 2.1. Study Design

A retrospective study was conducted by reviewing medical records of MMT patients to identify events of drop-out, death, and re-enrolment of MMT patients who registered in the Thai Nguyen MMT clinics from May 12, 2011 to September 6, 2015.

### 2.2. Subjects and Sample Size

All 2,638 medical records of patients who were registered in the Thai Nguyen MMT clinics from May 12, 2011, to September 6, 2015, were included in this study, provided they met the following inclusion criteria: (i) have been diagnosed with heroin addiction; (ii) complete information related to adherence and participation of patients. 2,567 eligible medical records were selected for extracting data related to his/her participation in the MMT program.

### 2.3. Study Variables

Variation of the MMT population consisted of the exiting (drop-out and death), the reenrolment, and the retention.

During treatment, patients who do not come to the MMT at health facilities on a daily basis are defined as “nonadherent patients” [5] and among these “nonadherent patients”, those absent for 30 consecutive days with or without reason after starting MMT are defined as “drop-out patients” [19, 29].

- Exiting was defined as drop-out patients plus patients who died while receiving MMT.
- Re-enrolment to the MMT was defined as patients who had received MMT but quit for at least 30 consecutive days and then returned to restart the MMT [5, 30].
- Retention in the MMT was defined as patients who were admitted to the MMT program in the period from May 12, 2011, to September 6, 2015, and still remained in the program up until the time of survey (September 6, 2015; a maximum of 53 follow-up months).
- To identify the risk of drop-out of the MMT by each year of participation, the cumulative drop-out rate at specific periods of treatment (the first, second, third, and fourth years of the MMT) was calculated

### 2.4. Data Collection and Analysis

Events related to the studied variables of drop-out, re-enrolment, and death during the MMT process were reviewed and entered into the data sheets designed in Excel by the dates that the events occurred. Data recorded in the Excel data sheet were cleaned and reentered and transferred to Stata 12 software for analysis. To measure the variation of the MMT population, the cumulative drop-out rate, cumulative death rate, and cumulative reenrolment rate were analyzed by applying Nelson-Aalen analysis techniques.

The equation of the Nelson-Aalen function which was used for calculating the cumulative drop-out, death, and re-enrolment rate is given by

(1) $$\begin{array}{c} {\tilde{H}}_{t}=\underset{t_{i}\leq t}{\sum }\frac{d_{i}}{n_{i}} \end{array}$$

t_i_ is the duration of study at point i, d_i_ is the number of events up to point i, and n_i_ is the number of individuals at risk just prior to t_i_. The cumulative hazard function H_hat (t) is the integral of the hazard rates from time 0 to t, which represents the accumulation of the hazard over time; mathematically this quantifies the number of times you would expect to see the failure event in a given time period, if the event was repeatable.

The rate of retention was estimated by using a proportion, in which the numerator was measured by number of patients registered in the MMT program during the study period at a specific point of time, with the numbers of those exiting the program (drop-out and death) subtracted and the numbers of reentering added. The denominator was defined by total cumulative admission of patients at that point in time.

The risk of drop-out or cumulative drop-out rate at a specific year of treatment was calculated by classifying patients into their years of the MMT at the point of time they still remained in the program. Within each strata, the cumulative drop-out rate was calculated by a proportion in which the numerator was defined by cumulative number of patients who quit the program at any point of time from the beginning of their registration to the end of the study period. The denominator was defined by total patients who had had that length of treatment.

### 2.5. Ethics

This study proposal was submitted to the Ethical Committee of the Vietnam Administration AIDS Committee (VAAC) and approved on No. 07/2015/NCKHCS.

## 3. Results

Table 1 shows the demographic characteristics of study patients. 2,567 patients who registered for MMT in Thai Nguyen from May 12, 2011, to September 6, 2015, were eligible for this study. Most of the patients (98.8%) were male and 80.2% were 30 to 49 years old. These 2,567 patients were distributed across the 6 MMT clinics, namely, Tuc Duyen, Pho Yen, Dai Tu, Dong Hy, Trung Thanh, and Phu Luong at 22.2%, 21.7%, 19.4%, 16.7%, 12.8%, and 7.4%, respectively.

**Figure 1:**
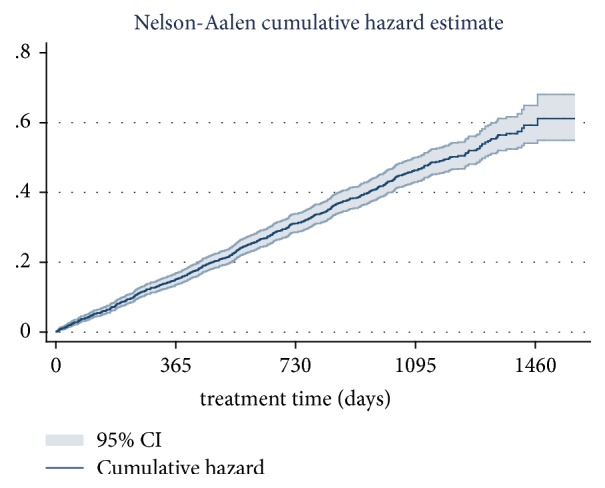
Cumulative hazard of exiting (quitting + death) of MMT program in every year.

Figure 1 reveals the cumulative hazard of exiting (include patients who dropped 30 consecutive days out of the MMT or died while receiving MMT during the 53 months of the study period). There was a gradual increase in the cumulative hazard of exiting over 53 follow-up months which were grouped into 4 periods at 0.15 (95% CI:0.13-0.17), 0.31 (95% CI: 0.28-0.34), 0.46 (95% CI: 0.43-0.50), and 0.61 (95% CI: 0.55-0.68), respectively.

**Figure 2:**
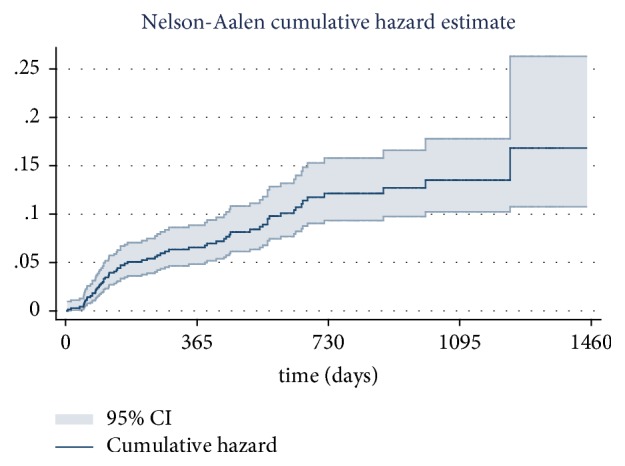
Cumulative hazard of re-enrolment in every year after quitting program in 740 patients.

Figure 2 shows the cumulative hazard of re-enrolment among 740 patients who quit the MMT program by specific follow-up years. 65 out of 740 patients returned to the MMT program after drop-out. The cumulative hazard of re-enrolment increased slightly from the first year to the fourth years after drop-out, with re-enrolment at 0.07 (95% CI: 0.05-0.09), 0.12 (95% CI: 0.09-0.16), 0.13 (95% CI: 0.10-0.18), and 0.16 (0.11-0.26), respectively. The number of patients who returned to the MMT program was especially high in the first and second years after drop-out.

**Figure 3:**
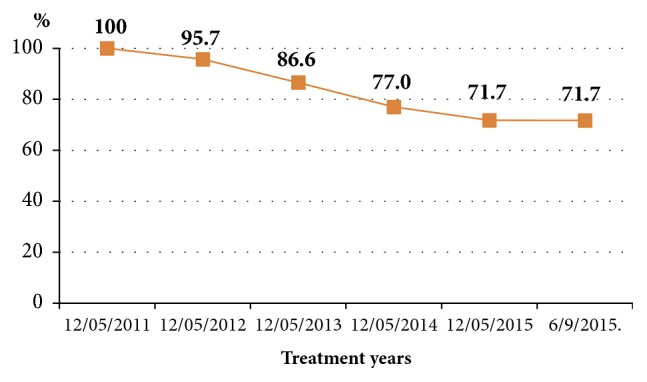
Rate of retention among 2,567 patients between 2011 and 2015.

Figure 3 illustrates the cumulative retention rate over 53 months of follow-up. There were 71.7% patients retained in the MMT program out of 2,567 patients who had registered in the MMT from May 12, 2011, to September 6, 2015, and had been followed up until September 6, 2015. The retention rate varied in a downward trend in the first 4 follow-up years from 100% to 71.7% and then kept stable afterwards.

**Figure 4:**
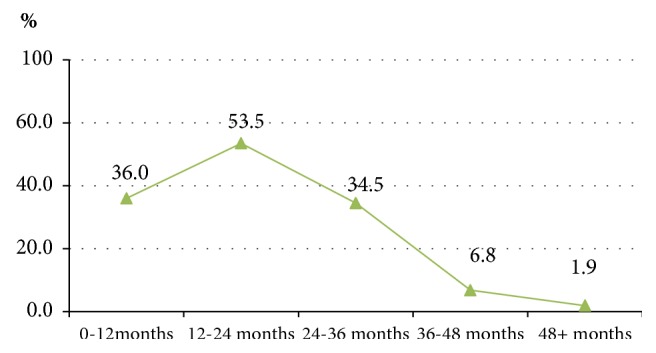
Risk of quitting rate by year of treatment.

Figure 4 presents the risk of drop-out of the MMT by years of MMT. By classifying patients into years of their MMT, this analysis tries to identify which year of MMT is likely to have the highest rate of drop-out. The risk of drop-out increased to 36.0% among those in their first year of MMT and then peaked at 53.5% among those in their second year of MMT. After that, the risk of drop-out decreased from the third year to fifth year.

## 4. Discussion

This study indicated that out of 2,567 patients who registered for the MMT from May 12, 2011, to September 6, 2015, 740 patients have quit and 52 patients have died during the 53 following months since the MMT program was established. The drop-out and death numbers constituted number of exiting the MMT and contributed to the variation of the MMT population within the MMT program. This current study indicated that the cumulative hazard of exiting increased over the studied period. Almost all studies which have investigated drop-out rates from MMT programs have analyzed drop-out over 7 continuous days' rate in a specific year and indicated that drop-out rate ranged from 30% to over 70% [9–11]. There are few studies focusing on analyzing trends of 30 continuous day dropping-out rate over longer period of MMT. However, there was a study in Ho Chi Minh City in Vietnam, which has analyzed dropping-out rate over time and showed that the MMT drop-out rate was 8.8% in 2009 and 10.9% in 2010 [25]. A similar study conducted by FHI in Ho Chi Minh City and Hai Phong indicated that 10.2% of patients have quit after one year and 17.7% have quit after two years of the MMT [26]. Findings of our study contribute to the previous findings the fact that the longer the MMT runs, the higher the risk of drop-out is. As a result, in the field of MMT management, in order to control drop-out cases over MMT algorithm, counseling adherence for patients not only at the beginning but also during the ongoing MMT is highly recommended.

Beside the factors of drop-out and death which contributed mostly to the variation of the MMT population, the number of reenrolling patients was also an issue of concern. This current study demonstrates that cumulative hazard of re-enrolment among 740 patients who quit the MMT program increased by every follow-up year of quitting. The re-enrolment hazard was the highest between the first and second year after the patients' drop-out and then increased slightly in the following years. The cumulative hazard of re-enrolment after 1 year of drop-out was 0.07 (95% CI: 0.05-0.09). The figures for 2 years, 3 years, and 4 years were 0.12 (95% CI: 0.09-0.16); 0.13 (95% CI: 0.10-0.18); 0.16 (0.11-0.26). In Vietnam, none of the existing studies evaluated the re-enrolment rate among patients who quit the MMT, but there were several studies in other countries focusing on this issue. The re-enrolment rate for the MMT programs among patients who dropped out ranged from 7.0% to 62.5% [7, 31]. Findings of the re-enrolment in this current study and previous studies brought a challenge to the MMT program that how to treat with these returning cases to control them from drop-out later. There was evidence that patients who attended two or more cognitive behavioral group sessions were more likely than those who attended 0-1 sessions or those in the comparison group to have returned to treatment during the 6-month follow-up time period (72 versus 53 versus 50%, respectively, p < 0.05, chi square test) [32]. As a result, special efforts on counseling should be reenforced for the re-enrolment patients of MMT.

The drop-out, death, and re-enrolment figures made the variation of the MMT population and these 3 events decided the retention rate which is one of indicators of the success of the MMT program [5, 7, 8]. This current study indicated that the cumulative rates of retention among 2,567 patients from 2011 to 2015 decreased rapidly in the first 3 years of running MMT program at 95.7%, 86.6%, and 77.0%, respectively, then slightly decreased in the fourth to 71.7%, and then remained stable in the fifth year. Overall, at the end of the 53 follow-up months of the MMT program, 28.3% of the patients were no longer in the program. A study in Yunnan, China, from March 2008 to February 2009 showed that the cumulative probability of retention dramatically decreased during the treatment period, with retention rates at 1 month, 3 months, and 6 months at 94.0%; 75.0%, and 57.0%, respectively [33]. A study among one-hundred and seventy-two outpatients in MMT clinics in China showed that the average retention rate was 94.8% at day 30 and 82.6% at day 90 [34]. Fingerhood, from the USA, indicated that, at 12 months, 61.9% of methadone patients were maintained in treatment [35]. Rhoades et al. showed that out of the 107 participants who completed stabilization and entered treatment, 71 (66.0%) were left at the end of the 24 weeks of the study [36]. A study in France (2014) indicated that the rate of retention during 12 months of treatment was 35.2% [37]. In Malaysia in 2012, patients were followed for 2 years and the rate of retention was 62.0% [11]. A study in an Outpatient Clinic in District 4, Ho Chi Minh City revealed that the retention rate after 2 years was 85.9% [38]. Hence, the rate of retention of patients in the MMT program in this study is similar to that of other studies in Vietnam but higher than some studies in the word.

Another issue that the current study focused on is identification of the specific point during MMT, as measured on a yearly basis, that patients have the highest risk of drop-out. The cumulative drop-out rate was the highest among patients who were classified as in the second year of the MMT process, at 53.5%. From the second year of the MMT process onwards, the drop-out rate gradually decreased. These findings indicate that MMT programs should offer more support for patients in the first 24 months of their MMT, as this is considered the most critical period with the highest drop-out rate. In the study evaluating MMT outcomes in an Atlantic Canadian milieu, the first year of MMT was indicated as critical period of drop-out of the MMT [39]. These evidences suggested that additional resources are needed during the first two year of treatment, given that these were the years of greatest change, but also greatest risk of drop-out from MMT. Therefore, patients and their families should be informed and consulted about the highest risk period of drop-out of the MMT in order to be prepared and plan to overcome challenges in this period.

## 5. Conclusions

There was a significant variation in the MMT population in Thai Nguyen from 2011 to 2015 given by drop-out, death, re-enrolment events. The cumulative hazard of exiting increased over the 53 months of the MMT program, with total of 740 patients having quit and 52 patients who died, while 65 patients reentered after drop-out. After 5 years of running the MMT program, 28.6% out of 2567 registered patients no longer remained in the MMT program. Patients who experience the second year of MMT have the highest risk of dropping-out the program. Therefore, patients and their families should be informed and consulted about the highest risk period of drop-out of the MMT and also about the time period when drop-out patients are most likely to reenter the MMT. Counseling adherence for patients which is conducted not only at the beginning but also during the ongoing MMT plays an extremely important role in reducing drop-out of the program while special counseling should also be reenforced for the re-enrolment patients of MMT.

## Figures and Tables

**Table 1:** Demographic characteristics of patients who registered for MMT from (May 12, 2011, to September 6, 2015) at the six Methadone Maintain Treatment clinics in Thai Nguyen.

**Characteristic**	**n**	%
**Gender**		
Male	2,536	98.8
Female	31	1.2
**Age group**		
≤29	257	10.0
30-49	2,058	80.2
≥50	252	9.8
**MMT clinics**		
Dong Hy	429	16.7
Dai Tu	498	19.4
Phu Luong	185	7.2
Pho Yen	558	21.7
Tuc Duyen	569	22.2
Trung Thanh	328	12.8
**Total**	**2,567**	**100**

## Data Availability

The Stata data used to support the findings of this study are currently under embargo by HMU and VAAC while the research findings are commercialized. Requests for data, 6 months after publication of this article, will be considered by the corresponding author and study groups.
